# Hepatic protection and anticancer activity of curcuma: A potential chemopreventive strategy against hepatocellular carcinoma

**DOI:** 10.3892/ijo.2013.2184

**Published:** 2013-11-21

**Authors:** YAN LI, XUE SHI, JINGWEN ZHANG, XIANG ZHANG, ROBERT C.G. MARTIN

**Affiliations:** 1 Divisions of Surgical Oncology, University of Louisville, Louisville, KY 40202, USA; 2 Gastroenterology/Hepatology, School of Medicine, University of Louisville, Louisville, KY 40202, USA; 3 Department of Chemistry, University of Louisville, Louisville, KY 40202, USA

**Keywords:** curcuma sesquiterpenoids, hepatocellular carcinoma and liver cancer

## Abstract

Malignant transformation of hepatocellular carcinoma (HCC) occurs through repetitive liver injury in a context of inflammation and oxidative DNA damage. A spectrum of natural sesquiterpenoids from curcuma oil has displayed anti-oxidant, anti-inflammatory and anti-carcinogenic properties. The aim of the study was to investigate the hepatoprotective and anti-HCC effects of curcuma oil *in vivo* and *in vitro*. Mice were pretreated with curcuma oil (100 mg/kg) for 3 days, then treated with Concanavalin A (30 mg/kg). The hepatic tissue was evaluated for histology, CD4^+^ cell, interferon-γ, apoptosis, lipid peroxidation, 8-hydroxy-deoxyguanosine and MnSOD. C57L/J mice were treated with curcuma oil and 10^7^ Hepa1-6 cells directly inoculated into liver lobes. The effects of curcuma oil on cell growth and cell death were evaluated. In addition, MnSOD, HSP60, catalase, NF-κB and caspase-3 were also investigated in the Hepa1-6 cells treated with curcuma oil. Pretreatment with curcuma oil significantly attenuates inflammation and oxidative damage by Concanavalin A. Treatment with curcuma oil can decrease the incidence of HCC. Curcuma oil inhibits cell growth and induces cell death in Hepa1-6 cells. Curcuma protected mice with hepatic injury from inflammatory and oxidative stress. Curcuma oil can inhibit hepatoma cell growth *in vivo* and *in vitro*.

## Introduction

Hepatocellular carcinoma (HCC) is the fifth most common malignant disorder worldwide and causes nearly one million deaths a year (1). Regardless of the etiological agent, malignant transformation of HCC is known to occur, in a context of inflammation and oxidative DNA damage (2). Inflammation and oxidative stress caused by hepatitis virus (HBV or HCV) infection play important roles in liver parenchyma fibrogenesis and carcinogenesis. It has been found that acute intracellular oxidative stress is usually elevated in chronic HCV patients who are at high risk of HCC (2), while transgenic mice expressing HCV core protein show an increased production of reactive oxygen species (ROS) (3,4). A correlation between oxidative stress and the transformation of HCC has also been suggested (5–7). All these studies lead to the hypothesis that an agent with anti-viral, anti-inflammatory and anti-oxidation attributes could contribute to prevention against HCC.

*Curcuma aromatica*, with multiple ingredients, has been identified as having anti-inflammatory, anti-oxidative stress and anticancer properties (8,9). Regarding the use of medicinal phytochemicals, the principal biological active components of *Curcuma aromatica* are consistent with two fractions: crystallization and volatile oil. The crystallization fraction is normally obtained by alcohol extraction, which mainly contains these related curcuminoids: curcumin, bisdesmethoxycurcumin and desmethoxycurcumin. The volatile fraction, extracted by steam distillation, contains a spectrum of related sesquiterpenoids including turmerone, germacrone and β-elemene (10).

At present, numerous*in vitro* studies have shown that curcumin exhibits a broad range of biological activities, including anti-carcinogenic effects (11); however, poor absorption and rapid elimination from the body raise questions about whether these biological activities actually occur *in vivo* (12). The other phytochemicals, especially the sesquiterpenoids in curcuma oil, in fact, have been demonstrated to show anticancer effect in some studies (13,14). Recently, turmerone, a major sesquiterpenoid in curcuma oil, has demonstrated both anti-inflammation and anticancer effects. Turmerone can not only suppress cyclooxygenase (COX-2) and nitric oxide synthase (iNOS), but also induce apoptosis in various cancer cell lines (15,16). In addition, turmerone showed immunomodulatory activities which could contribute to the anticancer effect (17). We have found that turmerone is rich in curcuma oil; however, studies are largely missing regarding the hepatopreventive and anti-HCC effects these substances. In this study, we investigated the hepatopreventive effect and anticancer effect of curcuma oil.

## Materials and methods

### Animals, cell line and materials

A University of Louisville Institutional Animal Care and Use Committee protocol #09101 was used for this study. Male 25–30-g C57 mice (Jackson Labs, Bar Harbor, ME, USA) were used in the Concanavalin A (Con A) liver injury model to evaluate properties of curcuma oil for anti-inflammation and anti-oxidative stress similar to our prior studies (18,19). Male 20–25-g, 8-week-old C57L/J mice (Jackson Labs) were used in the study of orthotopic inoculated hepatoma model to evaluate the possible anticancer effect of curcuma oil *in vivo*. Mouse hepatocellular carcinoma cell line Hepa1-6 was purchased from ATCC (Manassas, VA, USA). Curcuma essential oil (curcuma oil) was obtained from New Directions Aromatics Inc. (San Ramon, CA, USA). The ApopTag^®^ *In Situ* Apoptosis Detection kit was purchased from Intergen Co. (Purchase, NY, USA). Dako EnVision™+System kit was purchased from Dako Corp. (Carpinteria, CA, USA). OXItek TBARS assay kit was purchased from ZeptoMetrix Corp. (Buffalo, NY, USA). SOD assay kit-WST was purchased from Dojindo Molecular Technologies, Inc., (Gaithersburg, MD, USA). ALT (GPT) reagent kit was purchased from Thermo Electron Corp. (Waltham, MA, USA).

### The ingredient analysis of curcuma oil by GCxGC/TOF-MS

The molecular composition of curcuma oil was analyzed on a two-dimensional gas chromatography time-of-flight mass spectrometer (GCxGC/TOF-MS). Stock solutions were then transferred to GC vials waiting for analysis. The methoxymation and derivatization were prepared immediately before the GCxGC/TOF-MS analysis.

The Pegasus 4D GCxGC/TOF-MS instrument (LECO Corp., St. Joseph, MI, USA) was equipped with an Agilent 6890 gas chromatograph featuring a two-stage cryogenic modulator and secondary oven, 60 m × 0.25 mm i.d. × 0.25 *μ*m film thicknesses, DB-5ms GC capillary column [(5%-phenyl)-dimethylpolysiloxane, Agilent Technologies J&W] was used as the primary column for the GCxGC/TOF-MS analysis. A second GC column of 1 m × 0.25 mm i.d. × 0.25 *μ*m film thickness, DB-17ms [(50%-phenyl)-methylpolysiloxane, Agilent Technologies J&W] was placed inside the secondary GC oven after the thermal modulator.

LECO’s ChromaTOF software package (version 4.21) equipped with the National Institute of Standards and Technology (NIST) MS database (NIST MS Search 2.0, NIST/EPA/NIH Mass Spectral Library; NIST 2008) was used for instrument control, spectrum deconvolution and metabolite identification. We used the manufacturer’s recommended parameters for ChromaTOF to reduce the raw instrument data into a metabolite peak list. These parameters are: baseline offset, 1; smoothing, Auto; peak width in first dimension, 15 sec; peak width in the second dimension, 0.1 sec; signal-to-noise ratio (S/N), 10; match required to combine peaks, 700; R.T. shift, 0.08 sec; minimum forward similarity match before name is assigned, 600. The peak true spectrum was also exported as part of the information for each peak in absolute format of intensity values.

### Con A-induced hepatic injury model

Con A is a polyclonal mitogen which can induce T lymphocyte activation and a cytokine secretion syndrome that progressively destroys the liver parenchyma leading to organ-specific immune liver injury. We used this Con A liver injury model to evaluate the potential hepatoprotective effect of curcuma oil. Male C57 mice were divided into 3 groups. Con A + curcuma oil group was pretreated with curcuma oil at 100 mg/kg (i.p.) for 3 days followed by a treatment of Con A at 30 mg/kg (i.v., tail vein); Con A group was pretreated with saline (same volume as curcuma oil, i.p.) for 3 days and then treated with Con A at 30 mg/kg (i.v., tail vein); the control group was treated with saline only. Twelve hours after the administration of Con A, the mice were euthanized and liver tissue was harvested for the measurements of histology, ALT, apoptosis, lipid peroxidation and MnSOD enzymatic activity.

### Serum alanine aminotransferase activity

Alanine aminotransferase (ALT) enzymatic activity of serum samples was determined in liver tissues of all mice using an ALT (GPT) reagent kit according to the instructions provided.

### Orthotopic mouse liver cancer model

The orthotopic liver cancer model was established to evaluate the cancer incidence and the possible anti-HCC effect of curcuma oil. Hepa1-6 cell line is derivative of the BW7756 mouse hepatoma and shows reliable growth in the syngeneic host C57L/J mouse (17). Eight-week-old C57L/J mice were housed five per cage, given standard commercial chow (20) and tap water and maintained on a 12-h light/dark cycle. Hepa1-6 cells were maintained at 37°C, 95% air and 5% CO_2_ in Dulbecco’s modified Eagle’s medium (DMEM), supplemented with 10% FBS. The Hepa1-6 cells were injected into the right flank of C57L/J mice at 1×10^7^ in 100 *μ*l. When the tumors were reached a size of ∼1,000–2,000 mm^3^, the tumor cells were isolated for cell culture. The 3–4 generation of subcultured Hepa1-6 cells were used to established orthotopic growth liver cancer in C57L/J mice. The cells were directly inoculated into the left liver lobe of mice at 1×10^6^ through a 27-gauge needle. To prevent tumor cell leakage from the injection point, a gelatin sponge patch 2 mm in diameter was attached onto the site of injection for a few minutes after withdrawal of the needle. Twenty-five mice were divided into three groups, control group; tumor inoculation group; and tumor inoculation + curcuma oil group, rendomly. The mice were pretreated with curcuma oil every 3 day for 1 week before Hepa1-6 cell inoculation and treated curcuma oil every 3 day after Hepa1-6 cell inoculation, at does 100 mg/kg (i.p.). The same volume saline was administrated as controls. The curcuma dose was utilized as per our previous manuscripts and previous bioavailability studies (18,21).

### Histopathology, cancer incidence and tumor weight

The animals were sacrificed and entire liver were isolated and weighted. The entire liver lobes were removed to examine for gross abnormalities cancer incidence and histological studies in the Con A model and HCC model. Hematoxylin and eosin (H&E) and immunohistochemical staining were performed. Regarding the HCC model, appearance of tumor, intrahepatic invasion, peritoneal metastasis and tumor weight were evaluated. Tumor weight/liver weight was also calculated.

Terminal transferase-mediated dUTP nick-end-labeling (TUNEL) assay, immunohistochemical and immunofluorescent assays, thiobarbituric acid reactive substances (TBARS) assay and MnSOD enzymatic activity assay were all performed as per our prior studies.

### Assays for in vitro cell viability and apoptosis

Hepa1-6 cells were seeded in 96-well plates at a density of 2×10^5^ cells per well. Three days after cell seeding, the cells reached ∼80% confluence and were followed by serum-free medium for 12 h. The cells were then treated with curcuma oil that was dissolved in DMSO at the concentrations of 50, 100, 200 and 500 *μ*g for 2, 6, 12 and 24 h, respectively. After the treatment, the cells were evaluated for growth inhibition and apoptosis by curcuma oil.

Cell viability was evaluated by 3-(4,5-dimethyl-thiazol-2-yl)-2, 5-diphenyltetrazolium bromide (MTT) reduction assay. The mean optical density (OD) values from triplicate wells for each treatment were used as the index of cell viability.

For the TUNEL assay, plastic slide covers were precoated with a mixture of 0.01 mg/ml fibronectin, 0.03 mg/ml bovine collagen type I and 0.01 mg/ml bovine serum albumin and then inserted into 24-well plates. The cells were seeded on the slide cover in 24-well plates at a density of 5×10^5^ cells per well and then treated with curcuma oil at the same concentrations as the MTT reduction assay. The TUNEL-positive epithelial cells were counted against negative cells at a magnification, ×40. An apoptotic index was calculated.

### Immunofluorescent and immunohistochemical assays

Immunofluorescent assay was performed on the frozen section of liver tissues. The slides were dried and blocked with 10% serum in PBS-T. After washing, CD4 was stained by incubation with monoclonal anti-CD4 antibody labeled with fluorescein isothiocyanate (FITC). Interferon-γ (IFN-γ) was stained by monoclonal anti-IFN-γ antibody and rhodamine-conjugated donkey anti-mouse IgG. A negative control was included in each run. FITC-labeled CD4 and rhodamine-labeled IFN-γ were examined under a fluorescence microscope at the oil objective ×100 magnification.

### Western blot analysis

Western blot analysis was performed to determine the protein expression in the liver tissues as well as the cells. In brief, total protein of tissue or cells is isolated with a cold lysis buffer and total protein is determined spectrophotometrically (12).

### Statistical analysis

Student’s t-tests assuming unequal variance were performed. The results are expressed as mean values ± standard deviation. Comparisons were made among the treated and untreated control groups by analysis of variance. A p-value of <0.05 was considered statistically significant.

## Results

### The potential active ingredients and hepatic protection of curcuma oil

**Figure 1.**
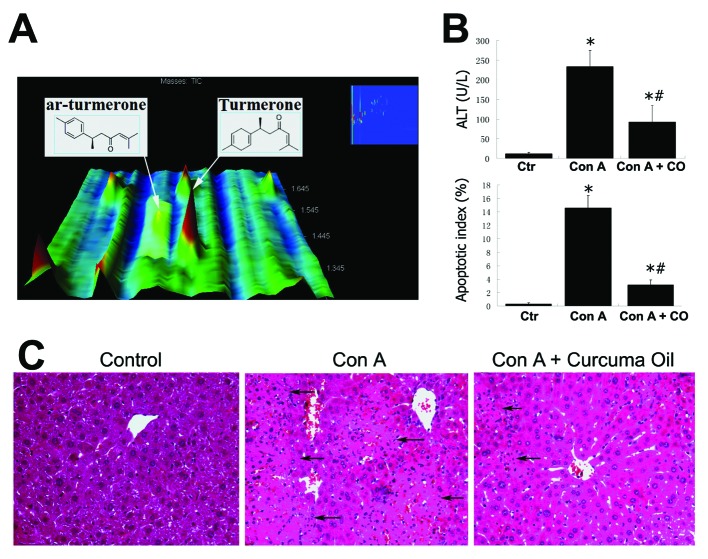
The potential active ingredients and hepatic protection of curcuma oil. (A) ar-turmerone eluted from the comprehensive GCxGC at 2154 sec in the first dimension column and 1.445 sec in the second dimension column with a peak area of 47342 and a spectrum similarity of 825. α-turmerone was detected at 2164 sec in the first dimension retention time and 1.365 sec in the second dimension retention time with a peak area of 146572 and a spectrum similarity of 859. The spectrum similarity ranges from 0 to 1,000. A high spectrum similarity indicates the high confidence of turmerone identification. (B) The effect of curcuma oil on alanine aminotransferase (ALT) level and apoptosis in hepatic tissue. Ctr, control group; Con A, Con A treated group; Con A + CO, curcuma oil pretreatment + Con A treated group. ^*^p<0.05 vs controls; ^#^p<0.05 vs Con A group. (C) Histological changes in hepatic tissue with Con A challenge. Arrows, necrosis. Hematoxylin and eosin staining (×200).

In total 909 ingredients were identified from the curcuma oil. Of the 909 ingredients, 105 are acids and most of them are organic acids. There are 252 kinds of alkenes, 243 alcohols, 133 ketos, 24 acetates and 9 oximes. Further analysis shows that 22 ingredients are considered as biologically active compounds. These ingredients include furanone, adamantaneacetic acid, fluorobenzoic acid, pyridinecarboxaldehyde, indazolinone, chlorobicyclo, acetamide, ascaridole epoxide, benzofuran, turmerone, etc. The biological activities of these compounds are various, for example, synthetic furanones have been shown to inhibit quorum-sensing and enhancing bacterial clearance in *Pseudomonas aeruginosa* lung infection in mice (22). Among these ingredients, turmerone is rich (Fig. 1A). Turmerone and ar-turmerone elute at different times from the two-dimensional GC because of the difference in their chemical structures.

Con A treatment induced severe hepatocytic injury and markedly increased serum ALT values at 233 U/l (p<0.01 compared to control) detected in Con A treated mice. However, pretreatment with curcuma oil attenuated the hepatocytic injury by Con A. The ALT level in the Con A + curcuma oil group was significantly decreased to 93 U/l (p<0.01 compared to Con A group) even though it was still elevated compared to 11 U/l in the control group (p<0.05). The increased apoptotic hepatocytes were also observed in the liver tissues by Con A challenge. The apoptotic index in the Con A group significantly increased in comparison to the controls (p<0.05). In the Con A + curcuma oil group, however, the apoptotic index was decreased statistically compared to that in the Con A challenged mice (p<0.05). The ALT levels and apoptotic index are shown in Fig. 1B. Microscopic examination by H&E stained liver sections revealed severe and extensive necrosis, acompanied by leukocyte infiltration and massive presence of red blood cells in the livers from Con A treated mice. This Con A induced hepatic injury was attenuated by the curcuma oil (Fig. 1C). Immunofluorescent detection revealed high CD4 positive staining in hepatic sinusoids and increased the early production of IFN-γ 12 h after Con A injection. While pretreatment with curcuma oil significantly attenuated the CD4 positive staining and production of IFN-γ in Con A challenged livers.

### Oxidative damage and hepatic protection of curcuma oil

**Figure 2.**
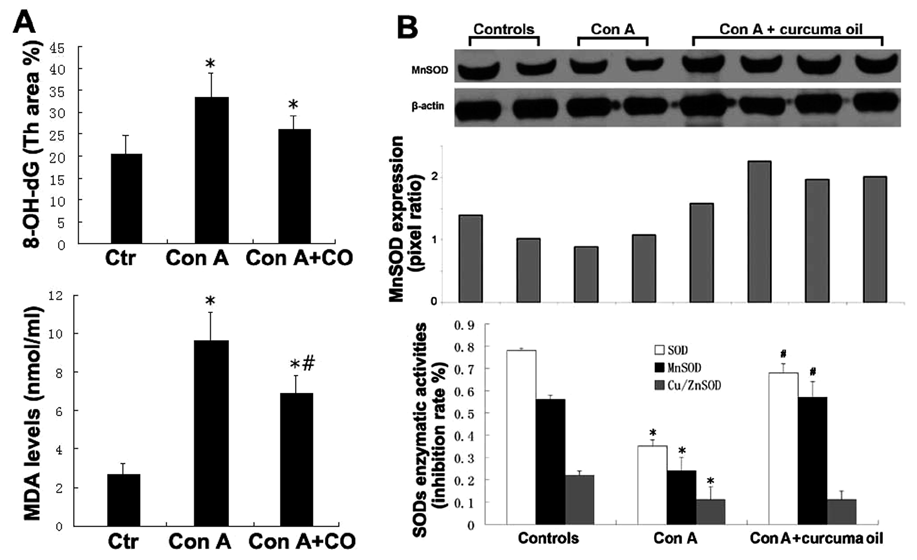
The oxidative damage and hepatic protection of curcuma oil. (A) Effect of curcuma oil on 8-OH-dG and lipid peroxidation in hepatic tissue. 8-OH-dG was detected by immunohistochemistry using computer image-analysis. Th area, the computer program quantified the threshold area represented by color images. Malondialdehyde (MDA) concentrations were measured representing the level of lipid peroxidation. Ctr, control group; Con A, Con A treated group; Con A + CO, curcuma oil pretreatment + Con A treated group. ^*^p<0.05 vs controls. ^#^p<0.05 vs Con A group. (B) The effect of curcuma oil on MnSOD protein expression and enzymatic activity. MnSOD expression was determined by western blotting and the optical density was further quantified by computer imaging software. Pixel ratio (MnSOD/β-actin) was used as the MnSOD expression levels. Enzymatic activities of SODs were determined by a colorimetric assay. SOD, total SOD enzymatic activity; MnSOD, MnSOD enzymatic activity; Cu/ZnSOD; Cu/ZnSOD enzymatic activity. CS, ^*^p<0.05 vs controls. ^#^p<0.05 vs Con A group.

Oxidative damage is implicated in the progression of acute inflammatory liver injury to chronic inflammatory liver disease. In this study, we performed the measurements regarding oxidative damage by Con A challenged liver injury. 8-OH-dG was measured for DNA oxidative damage and TBARS was measured for lipid peroxidation. The levels of 8-OH-dG in the hepatic tissues were evaluated by immunohistochemical staining along with computer image analysis. As compared with the controls, the level of 8-OH-dG was significantly increased in the hepatic tissues by Con A challenge. However, the level of 8-OH-dG was significantly decreased in the hepatic tissues of mice pretreated with curcuma oil. 8-OH-dG levels were not significantly different between the curcuma oil + Con A group and the saline controls. The TBARS results indicated that the level of MDA in the hepatic tissues of Con A challenged mice was considerably increased compared to saline controls. However, pretreatment with curcuma oil resulted in a significantly decreased lipid peroxidation product in the hepatic tissues after Con A challenge. The results of 8-OH-dG and TBARS are shown in Fig. 2A.

MnSOD expression and SODs enzymatic activity were also determined in the hepatic tissues of each sample. The results indicated that the level of MnSOD expression decreased after Con A administration. Pretreatment with curcuma oil attenuated the decreased level of MnSOD in the Con A affected hepatic tissue. Regarding SOD enzymatic activities, there was a significant loss of total SOD activity and MnSOD activity in the hepatic tissues by Con A challenging compared to the controls. The loss of both MnSOD activity and Cu/ZnSOD contributes to the decreased level of total SOD. Treatment with curcuma oil can significantly increase MnSOD activity, but not the Cu/ZnSOD level. The results of MnSOD expression and MnSOD enzymatic activities are shown in Fig. 2B.

### Curcuma oil inhibits hepatoma cell growth in the orthotopic inoculated model

**Figure 3.**
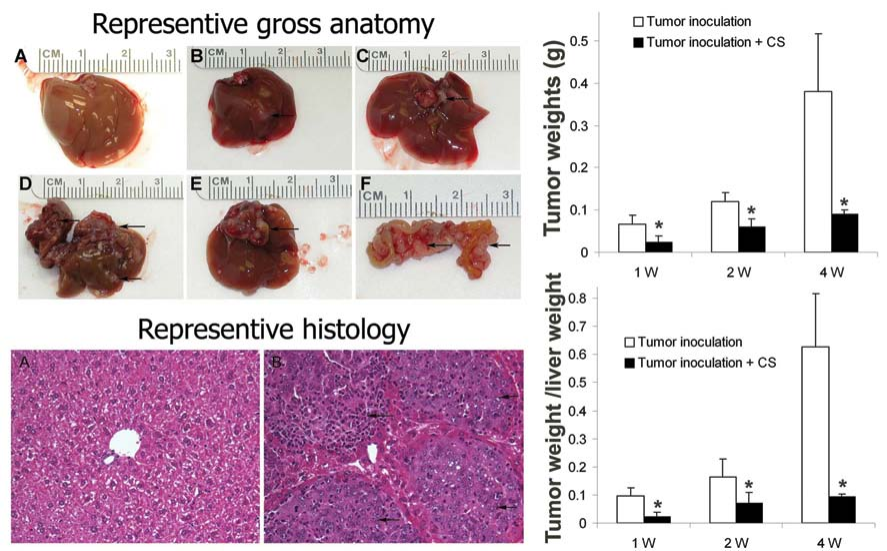
Representative figures of liver cancer and tumor weight. Left upper, representative images of gross anatomy of the liver cancer model. (A) Normal liver. (B) Tumor growth in the lobe of inoculation. (C) Tumor cells grow in site other than original inoculation. (D and E) Tumor invaded into the other lobes. (F) Inoculated tumor metastasized into intestinal mesenterium. Left bottom, representative histology of the liver cancer model. (A) Normal liver. (B) Liver cancer. Arrows, inoculated tumor cells. Hematoxylin and eosin staining (×200). Right upper, tumor weights in Hepa1-6 cell inoculation and in Hepa1-6 cell inoculation with pretreatment of curcuma sesquiterpenoids. Right bottom, tumor weight/liver weight in Hepa1-6 cell inoculation and in Hepa1-6 cell inoculation with pretreatment of curcuma sesquiterpenoids. CS, curcuma sesquiterpenoids. W, week. ^*^p<0.05 vs controls.

We evaluated the effect of curcuma oil on the inoculated tumor cell growth in an orthotopic HCC mouse model. Tumor weight, tumor intrahepatic distribution and tumor peritoneal metastasis were evaluated. The results indicated that all animals with a hepatoma cell inoculation were found with liver tumor, however the tumor weight was reduced in the hepatoma cell inoculated mice pretreated with curcuma oil. The intrahepatic metastasis and peritoneal metastasis were also inhibited in the curcuma oil treated group. The intrahepatic distribution and peritoneal metastasis in the tumor cell inoculation group and in the curcuma oil treated group at 1, 2 and 4 weeks are shown in Table I. Representive images of gross anatomy, histology by H&E staining, tumor weight and tumor weight/liver weight are shown in Fig. 3.

### The effects of curcuma oil on hepatoma cells in vitro

**Figure 4.**
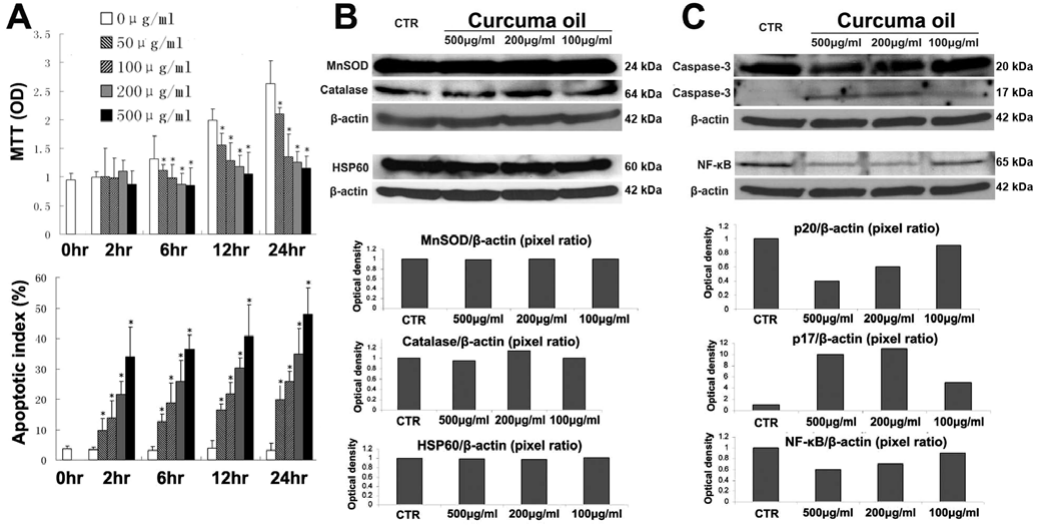
The effects of curcuma oil on hepatoma cells *in vitro*. (A) Curcuma oil inhibits growth and induces apoptosis in hepatoma cells. Hepa1-6 cells were seeded in a 96-well plate at 2×10^5^ cells/well. Curcuma sesquiterpenoids were added at the concentrations 50, 100, 200 and 500 *μ*g/ml for 2, 6, 12 and 24 h. MTT assay was used to determine cell viability. Spectrophotometric OD value was used as the cell growth index. TUNEL assay was used to determine cell apoptosis. Results are presented as the percentage of apoptotic cells over total cells. The values are expressed as means ± SD. ^*^p<0.05 vs untreated cells (0 *μ*g/ml). (B and C) Effect of curcuma oil on MnSOD, catalase, HSP60, caspase-3 and NF-^κ^B in Hepa1-6 cells. Hepa1-6 cells were seeded in 100-mm dish at 1×10^6^ cells and reached 95% confluence. The cells were treated with curcuma oil at the concentrations 100, 200 and 500 *μ*g/ml for 2 h. After treatment, total protein was extracted to perform the western blotting. The optical density was further quantified by computer imaging software and the pixel ratio was used as the expression levels. CTR, control (untreated).

The cell viability results indicated that curcuma oil can significantly inhibit the Hepl-6 cell growth. The inhibition of Hepa1-6 cell growth by curcuma oil showed a dose/time-effect relationship. We also investigated the effect of curcuma oil on cell apoptosis using a TUNEL assay. The results indicated that curcuma oil can also induce Hepal-6 cell apoptosis in a dose/time-effect manner (Fig. 4A).

We have found that curcuma oil can increase MnSOD protein level and enzymatic activity against Con A insults. If the MnSOD protein level and enzymatic activity were enhanced by curcuma oil in the cancer cells, the increased oxidative defense may favor cell growth. Therefore, we further investigated oxidative defense related enzymes such as MnSOD and catalase and stress response proteins such as heat shock protein 60 (HSP60). As opposed to findings in the normal hepatocytes, curcuma oil did not alter the MnSOD, catalase or HS60 in the Hepa1-6 cells (Fig. 4B).

We found that curcuma oil can induce apoptosis in Hepa1-6 cells. The measurement of a critical apoptotic effector, caspase-3, indicated that curcuma oil can significantly affect the apoptotic effector protein level. The caspase-3 precursor, p20 peptide, is decreased while the active caspase-3 enzyme, p17 subunit is generated after curcuma oil treatment. The NF-κB protein in response to the curcuma oil showed that NF-κB protein was decreased after curcuma oil administration (Fig. 4C).

**Figure 5.**
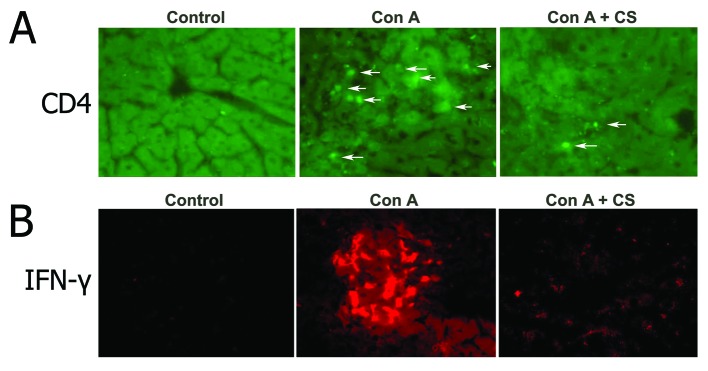
Effect of curcuma sesquiterpenoids on CD4^+^ T-cells and IFN-γ in hepatic tissue with Con A challenge. (A) FITC-immunofluorescence detection of CD4^+^ T-cells (×200). (B) Rhodamine-immunofluorescence detection of IFN-γ. Arrows, CD4 positive cells. CS, curcuma sesquiterpenoids.

To investigate whether curcuma oil could affect the Con A caused inflammatory changes, we performed measurements of CD4^+^ T-cells and IFN-γ. The results indicated that curcuma oil can decrease the CD4^+^ T-cell infiltration in hepatic parenchyma and the production of IFN-γ in the Con A challenged hepatic tissue (Fig. 5).

## Discussion

Two animal models are used in this study are: i) hepatitis by Con A and ii) liver cancer by hepatoma cell implantation. Con A induced hepatic tissue injury through activation of T lymphocytes and related cytokines is similar to tissue injury in chronic hepatitis of viral origin (HVB and HVC) and cirrhosis, which are high risk factors for HCC carcinogenesis. The established orthotopic mouse HCC model is feasible because Hepa1-6 cells is a mouse hepatoma cell line and it has been demonstrated that Hepa1-6 cells can grow in C57L/J mouse with the potential of metastasis (23). We used these two models to study curcuma oil regarding anti-inflammation, anti-oxidation and anticancer properties. In addition to animal models, we also performed an *in vitro* study of Hepa1-6 cells to investigate the effects of growth inhibition and apoptosis by curcuma oil.

Curcuma oil, consisting of multiple sesquiterpenoids, is steam-distilled from the rhizome of *Curcuma longa*. Although curcumin has been found as an important bioactive compound in *Curcuma longa*, poor systemic absorption of curcumin was also reported (24,25). Recently, curcuma sesquiterpenoids from the volatile oil fraction have been found with similar bioactivities to curcumin. Some sesquiterpenoids with anticancer activity have been identified such as turmerone and β-elemene (26,27). Unlike curcumin, sesquiterpenoids receive less attention and the precise molecular mechanisms underlying their bioactivities are largely unknown. We investigated the effects of curcuma oil (multiple sesquiterpenoids) on hepatic parenchyma protection and HCC chemoprevention.

Intravenous administration of Con A is an excellent model because it would resemble hepatic injury, with inflammatory cells infiltrating into the hepatic parenchyma and triggering hepatocyte death. We used a Con A model to study the hepatoprotective effect of curcuma oil. Our result indicated that Con A induces severe liver injury including elevation of ALT, apoptosis and necrosis, which is similar to other previous reports (28,29). Pretreatment with curcuma oil can attenuate Con A-induced ALT elevation and cell death. There are several lines of evidence showing anti-inflammatory properties of curcuma sesquiterpenoids. These include inhibition of nitric oxide (NO) production in LPS-activated macrophages by sesquiterpenoids from curcuma (30); inhibition of chemokines, COX-2 and receptor activator of nuclear factor-κB (NF-κB) ligand (RANKL)(31); and inhibition of paw swelling, COX-2 activity and serum haptoglobin in an adjuvant arthritic mouse model using curcuma extract (32). Our previous study showed that curcuma oil can significantly inhibit esophagitis induced by duodenogastroesophageal reflux (11). Therefore, the hepatic protection by curcuma oil could be through anti-inflammatory properties.

The results of CD4^+^ T-cells and IFN-γ (Fig. 5) indicated that curcuma oil can decrease the CD4^+^ T-cell infiltration in hepatic parenchyma and the production of IFN-γ in the Con A challenged hepatic tissue. As well known, Con A-induced liver injury is mediated by the activation of innate and adaptive immune cells, including NK cells, Kupffer cells and CD4^+^ T-cells and their production of inflammatory cytokines such as IFN-γ. It has been demonstrated the IFN-γ plays a critical role in T-cell-dependent liver injury initiated by Con A. The decreased levels of CD4^+^ T cells and IFN-γ might be a potential protective mechanism of curcuma oil against Con A-induced hepatic injury. This hypothesis is supported by the following observations. i) Pretreatment with monoclonal anti-mouse CD4 antibodies fully protected mice against Con A-induced hepatic injury (33); ii) Mice with severe combined immunodeficiency syndrome as well as athymic nude mice were resistant against Con A (34); and iii) IFN-γ knockout mice are protected from hepatic injury by Con A (35). Taken collectively, the anti-inflammatory effect of curcuma oil could be attributable, at least partly, to inhibition of CD4^+^ T lymphocytes migration and the production of IFN-γ, thereby protecting the hepatic cells against Con A insults.

The protective effect of curcuma oil against Con A could be also the potential activity of free radical scavenging, which has been demonstrated in other studies (14,36). We further explored the role of curcuma oil against oxidative damage. The results indicated that curcuma oil can not only decrease the levels of 8-OH-dG and lipid peroxidation, but also increase the protein level and enzymatic activity of MnSOD in the Con A challenged hepatic tissues. It has been demonstrated that sesquiterpenoids in curcuma species play important role as potent anti-oxidant (37). Interestingly, curcumin is poorly absorbed following ingestion, however diet supplementation with *Curcuma longa* or the whole extract of *Curcuma longa* to animals still showed antioxidant activity (9,38,39). This implicated that the sesquiterpenoids existing in *Curcuma longa* could possess the anti-oxidative activities and sesquiterpenoids could be more bioavailable than curcumin. We have demonstrated that curcuma oil can significantly decrease the levels of both lipid peroxidation and 8-OH-dG in the esophageal epithelium damaged by esophagoduodenal anastomosis induced reflux (11). Evidence is also provided by other studies, which include: i) supplementation with *Curcuma longa* reduces oxidative stress and lowers plasma lipid peroxide in rabbits fed a high cholesterol diet (9); ii) a significant reversal in lipid peroxidation, brain lipids and produced enhancement of glutathione by *Curcuma aromatica* was observed in model of brain injury with ethanol intoxicated rats (40). Therefore, the antioxidant activity could be from the sesquiterpenoids of curcuma. The potential antioxidant activity of curcuma oil could contribute to the hepatoprotection against oxidative stress-mediated destruction of hepatic parenchymal cells.

Actually, the active sesquiterpenoids in curcuma have been studied, while a spectrum of anticancer ingredients such as turmerone and β-elemene has enhanced our knowledge on the biologic functions of curcuma oil against cancer (41). Curcuma oil was found to exhibit an anti-proliferative effect in HepG2 cells by inducing apoptosis (13). This growth inhibition of curcuma oil is associated with cell cycle arrest, cytochrome C translocation, caspase-3 activation, poly-ADP-ribose polymerase (PARP) degradation and loss of mitochondrial membrane potential (13). The inhibitory effects of curcuma oil on hepatoma *in vivo* were also found and the growth inhibition by curcuma oil on hepatoma in mice might be associated with its depression on cellular proliferative activity (42). Our recent study showed that curcuma oil can prevent the carcinogenetic transformation of esophageal adenocarcinoma (11). The result in this study is in agreement with the above observations. We found that curcuma oil can inhibit the tumor cell growth both *in vivo* and *in vitro*. Interestingly, we found that treatment with curcuma oil did not change the protein levels of MnSOD, catalase and HSP60 *in vitro*, but the apoptotic effector (caspase-3) was activated. As we know, the caspase-3 precursor is first cleaved at Asp 175-Ser 176 to produce the p11 subunit and the p20 peptide. Subsequently, the p20 peptide is cleaved at Asp 28-Ser 29 to generate the mature p17 subunit. Our result indicated a decreased p20 peptide but increased p17 subunit. The p17 peptide is an important active caspase-3 subunit during the execution of the apoptotic cascade. The increased p17 subunit level of caspase-3 implied that curcuma oil contributes to cell apoptosis. The mechanism needs to be further explored. NF-κB has been proposed to be involved in the regulation of genes which control apoptotic cell death. Activation of NF-κB in cancer cells is correlated with the inhibition of apoptosis that leads to increased expression of anti-apoptotic proteins (43). We found that curcuma oil decreased NF-κB expression in Hepa1-6 cells. Although decreased NF-κB expression is consistent with increased apoptosis, the NF-κB DNA binding activity and the potential signaling of the regulation for anti-apoptotic proteins need to be further investigated.

Two important issues of curcuma oil addressed in this study are hepatoprotection and inhibition of HCC. However, it should be noted that some limitations exist. Con A-induced hepatocyte injury and liver lobe inoculation of hepatoma cells are adequate for evaluation of the bioactivities of curcuma oil regarding anti-inflammation, anti-oxidation and antitumor effects, but use of these two animal models is limited. A model of transformation from normal to hepatitis to HCC, such as diethylnitrosamine-induced liver cancer, is needed to provide a connection between hepatic injury and HCC carcinogenesis, thereby to determine the chemopreventive and chemotherapeutic effects of curcuma sesquiterpenoids. A study of combination of diethylnitrosamine and partial hepatectomy-induced liver cancer is underway to provide further insights into the effects of curcuma oil on HCC transformation. In this study, we only focus on the effects of hepatoprotection and HCC inhibition by curcuma oil *in vivo*; the mechanisms for hepatoprotection and HCC inhibition, especially the pharmaceutical targets of curcuma oil, need to be further investigated both *in vivo* and *in vitro*.

In conclusion, curcuma oil shows promising properties for hepatoprotection in Con A-induced injury and for chemotherapeutic effect against inoculated hepatoma in mice. It retains anti-inflammation, anti-oxidation and antitumor properties while adding potential advantages: multiple target effects and fewer side effects. Furthermore, it may be useful for hepatoprotection, HCC chemoprevention and for cancer patients as a long-term maintenance drug to prevent tumor recurrence.

## Figures and Tables

**Table I.** Incidence of HCC in mice with Hepa1-6 cell liver orthotopic implantation.

		1 week			2 weeks			3 weeks		
		
	n	TN	HD	PM	TN	HD	PM	TN	HD	PM
Controls	5							0 (5)	0 (5)	0 (5)
Tumor inoculation	10	3 (3)	1 (3)	0 (3)	3 (3)	2 (3)	2 (3)	4 (4)	4 (4)	4 (4)
Tumor inoculation + CS	10	3 (3)	0 (3)	0 (3)	3 (3)	1 (3)	1 (3)	4 (4)	2 (4)	2 (4)

TN, tumor number; HD, hepatic distribution; PM, peritoneal metastasis; CS, curcuma sesquiterpenoids.
